# Activation of formate hydrogen-lyase via expression of uptake [NiFe]-hydrogenase in *Escherichia coli* BL21(DE3)

**DOI:** 10.1186/s12934-015-0343-0

**Published:** 2015-09-22

**Authors:** Byung Hoon Jo, Hyung Joon Cha

**Affiliations:** Department of Chemical Engineering, Pohang University of Science and Technology, Pohang, 790-784 Korea

**Keywords:** Recombinant hydrogenase, *Escherichia coli* BL21(DE3), Biohydrogen, Formate hydrogen lyase, *Hydrogenovibrio marinus*

## Abstract

**Background:**

Several recent studies have reported successful hydrogen (H_2_) production achieved via recombinant expression of uptake [NiFe]-hydrogenases from *Hydrogenovibrio marinus*, *Rhodobacter sphaeroides*, and *Escherichia coli* (hydrogenase-1) in *E. coli* BL21(DE3), a strain that lacks H_2_-evolving activity. However, there are some unclear points that do not support the conclusion that the recombinant hydrogenases are responsible for the in vivo H_2_ production.

**Results:**

Unlike wild-type BL21(DE3), the recombinant BL21(DE3) strains possessed formate hydrogen-lyase (FHL) activities. Through experiments using *fdhF* (formate dehydrogenase-H) or *hycE* (hydrogenase-3) mutants, it was shown that H_2_ production was almost exclusively dependent on FHL. Upon expression of hydrogenase, extracellular formate concentration was changed even in the mutant strains lacking FHL, indicating that formate metabolism other than FHL was also affected. The two subunits of *H. marinus* uptake [NiFe]-hydrogenase could activate FHL independently of each other, implying the presence of more than two different mechanisms for FHL activation in BL21(DE3). It was also revealed that the signal peptide in the small subunit was essential for activation of FHL via the small subunit.

**Conclusions:**

Herein, we demonstrated that the production of H_2_ was indeed induced via native FHL activated by the expression of recombinant hydrogenases. The recombinant strains with [NiFe]-hydrogenase appear to be unsuitable for practical in vivo H_2_ production due to their relatively low H_2_ yields and productivities. We suggest that an improved H_2_-producing cell factory could be designed by constructing a well characterized and overproduced synthetic H_2_ pathway and fully activating the native FHL in BL21(DE3).

## Background

Hydrogen (H_2_) production via biological means has been considered as a potential source of alternative fuel due to clean and truly renewable processes [1]. Hydrogenases are the key enzymes in microbial H_2_ metabolism that catalyze the reversible reduction of protons with electrons [2]. Certain limitations of native hydrogenase systems for H_2_ production (i.e., problems related to substrate (electron donor/acceptor) specificity, oxygen (O_2_) sensitivity, catalytic bias to H_2_ oxidation, electron partitioning, etc.) have been reported in microorganisms [3], and their properties appear to be unable to meet current needs. Therefore, expression and engineering of hydrogenases in heterologous hosts is generally accepted as the most influential approach to modification of enzyme qualities and H_2_ production efficiency for biotechnological applications [3, 4]. Recombinant expression of hydrogenase not only provides the ability to engineer the H_2_ metabolism of the host for specific purposes but also could facilitate basic studies on the maturation process of the complex metalloenzyme [4].

*Escherichia coli* has been widely used as a host microbe for protein expression [5]. This bacterium was also adopted for expression of recombinant hydrogenase in several studies, either for study of hydrogenase maturation or for improvement of fermentative H_2_ production by coupling to the native electron transfer system of *E. coli* [6–10]. In particular, the strain BL21(DE3) (or BL21), which is an optimized host for protein overexpression, can neither produce nor consume H_2_ (no hydrogenase activity) under the general culture conditions where K-12 derivatives do possess the abilities [11–14]. This observation prompted certain researchers to consider this strain as an ideal host for hydrogenase expression and testing for in vivo H_2_ production [12–14].

According to the composition of bimetallic active sites, hydrogenases are broadly classified into [FeFe]- and [NiFe]-hydrogenases from the standpoint of biotechnological importance. *E. coli* contains four different [NiFe]-hydrogenases, and among those, hydrogenase-3 is responsible for H_2_ production during mixed-acid fermentation [15]. This enzyme forms a formate hydrogen-lyase (FHL) complex together with formate dehydrogenase-H, one of the three formate dehydrogenases of *E. coli* [16].

Recently, certain studies reported that homologous or heterologous expression of the structural (large and small) subunits of uptake [NiFe]-hydrogenases resulted in construction of recombinant BL21(DE3) derivatives that are capable of producing H_2_ [17–19]. However, some unclear points arise that do not support the conclusion that the expressed hydrogenases are indeed responsible for the in vivo H_2_ production of the recombinant strains. Among these points, the most critical is that all of the engineered hydrogenases engage in H_2_ uptake (consumption) and not production in their native hosts [20–22]. In this work, we tackle this problem using simple biochemical and mutant experiments. We suggest that H_2_ production in such recombinant systems is almost exclusively dependent on the native FHL of *E. coli*, and thus, careful characterization of the recombinant hydrogenase systems in BL21(DE3) is required, especially for those designed for in vivo H_2_ production.

## Results and discussion

### Activation of FHL activity in recombinant strains

Several efforts have been put forth to engineer uptake [NiFe]-hydrogenases in BL21(DE3) strain [17–19]. In these studies, H_2_ production was demonstrated by expressing structural (large and small) subunits of the hydrogenases in the non-H_2_ producing *E. coli* strain, and the authors concluded that the engineered, non-native hydrogenases could be used as tools to enhance biohydrogen production in *E. coli*. However, a critical discussion promptly arises related to the fundamental origin of the produced H_2_: (1) The engineered hydrogenases are engaged in H_2_ uptake and not in H_2_ production in their native hosts, which means that standard redox potentials of their respective electron acceptors (e.g., cytochrome *b*) are expected to be much higher than that of H_2_ (−420 mV) [23]. Additionally, uptake [NiFe]-hydrogenases generally show high catalytic bias to H_2_ oxidation [24, 25]. Thus, even if an uptake [NiFe]-hydrogenase is ‘wired’ to an electron transport system in *E. coli*, H_2_ produced via the non-native pathway is not expected to highly accumulate in a closed batch culture system [12], which is in contrast to the results of high H_2_ accumulation in the previous studies [17–19]. (2) Addition of hypophosphite, an inhibitor of pyruvate formate-lyase, abolished the H_2_ production in a recombinant strain expressing *E. coli* HyaBA (hydrogenase-1) [19]. Moreover, addition of formate greatly increased in vivo H_2_ production. (3) Full maturation of the expressed hydrogenases is questionable because maturation of [NiFe]-hydrogenase further requires highly specific auxiliary proteins [26].

**Fig. 1:**
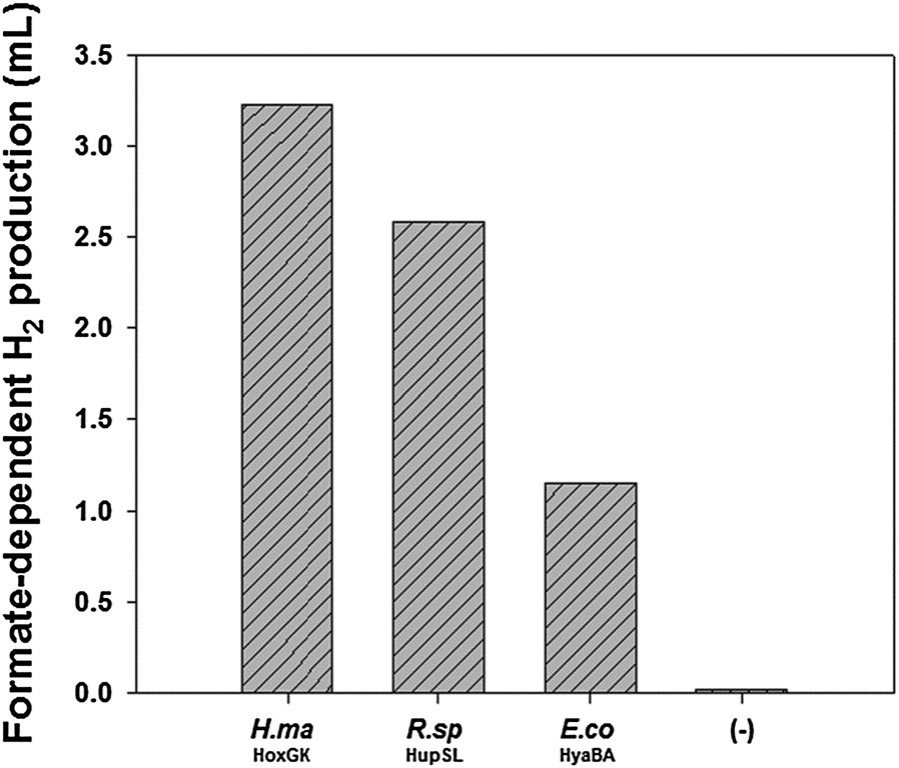
FHL activation in *E. coli* BL21(DE3). Recombinant cells harboring each hydrogenase were cultured in PBS buffer supplemented with 20 mM sodium formate, and H_2_ production from formate was measured after 13 h. *H.ma*, *Hydrogenovibrio marinus*; *R.sp*, *Rhodobacter sphaeroides*; *E.co*, *Escherichia coli*; (−), negative control strain with parental empty vector (pTrcHis C)

Putting the theoretical and the experimental clues together, we hypothesized that the BL21(DE3) derivatives produce H_2_ via a native FHL pathway that is activated by the expression of the recombinant hydrogenases. A test for H_2_ production using formate as a sole electron source showed that the recombinant strains with the heterologous (*H. marinus* HoxGK and *R. sphaeroides* HupSL) or homologous (*E. coli* HyaBA) hydrogenase indeed showed FHL activity, whereas the negative control strain with the parental empty vector exhibited negligible FHL activity as expected (Fig. 1). When we measured formate consumption by the strain with *H. marinus* HoxGK, it was found that the cells consumed 1.6 ± 0.1 mM formate, whose corresponding calculated H_2_ production (3.58 mL) well coincides with the actual amount of H_2_ production (3.23 mL). In contrast, the negative control cells showed virtually no consumption of formate (0.0 ± 0.1 mM). These results imply that the FHL pathway was at least partially responsible for the observed in vivo H_2_ production in the previously reported recombinant strains.

### FHL dependency of H_2_ production in the recombinant strains

Measurement of FHL activity was not sufficient to decide whether H_2_ production in the recombinant strains originates exclusively from the activated FHL pathway. To examine the FHL-dependency, we constructed two knockout BL21(DE3) strains lacking formate dehydrogenase-H (*fdhF*) and hydrogenase-3 (*hycE*), respectively, both of which constitute essential components of the FHL complex [16] and subsequently tested in vivo H_2_ production by expressing the recombinant hydrogenases.

**Fig. 2:**
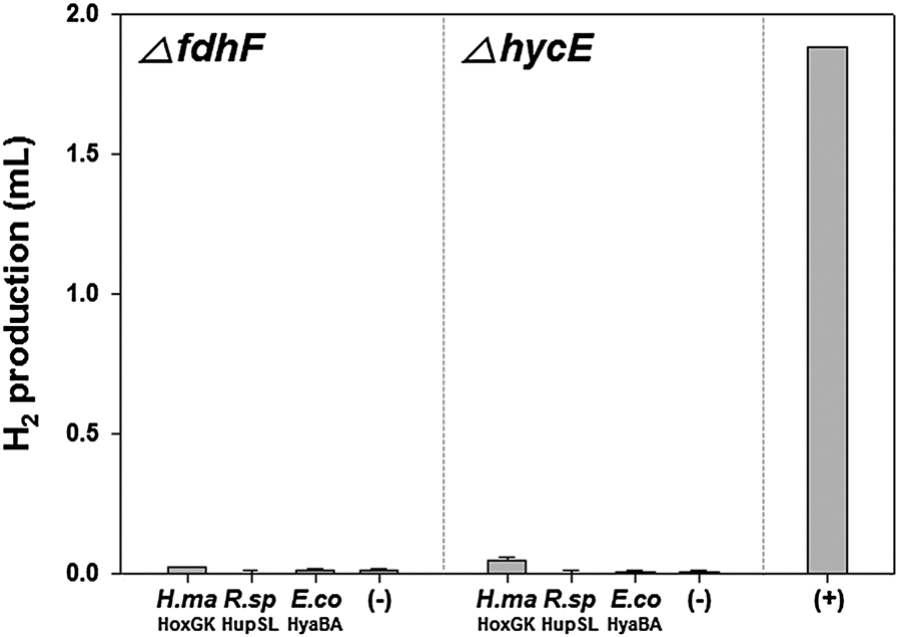
H_2_ production in FHL-deficient mutant BL21(DE3) strains. Strains lacking formate dehydrogenase-H or hydrogenase-3 were used. *H.ma*, *Hydrogenovibrio marinus*; *R.sp*, *Rhodobacter sphaeroides*; *E.co*, *Escherichia coli*; (−), negative control mutant with parental empty vector (pTrcHis C); (+), positive control strain with *R. sphaeroides* HupSL

In the case of the *fdhF* mutant, all mutant strains produced small amounts of H_2_ that were roughly comparable to that of the negative control (Fig. 2), which clearly demonstrated that H_2_ was produced from formate as the only major substrate in the previous reported recombinant strains [17–19]. Similarly, insignificant amounts of H_2_ were produced by *hycE* mutants, which indicates that hydrogenase-3 was almost entirely responsible for H_2_ production in the reported BL21(DE3) derivatives (Fig. 2). Although H_2_ production by both of the mutants with *H. marinus* HoxGK was slightly exceptional (2.1-fold for *fdhF* mutant and 7.5-fold for *hycE* mutant compared with the negative controls), the amounts can still be considered marginal compared with the positive control. It appears that the expression of HoxGK influenced the other *E. coli* hydrogenase system(s) to evolve H_2_ because H_2_ was not detected when the *E. coli* MW1001 strain lacking hydrogenase-1, hydrogenase-2, and hydrogenase-3 was transformed with pTrcHoxGK (data not shown). Thus, we concluded that H_2_ was produced almost exclusively via the activated FHL pathway in the BL21(DE3) strains with the recombinant hydrogenases. We strongly suspect that the recent report on H_2_ production in BL21(DE3) by expression of *Rhodopseudomonas palustris* [NiFe]-hydrogenase [27] falls within this category. It is noteworthy that all recombinant [NiFe]-hydrogenases that activated FHL belong to Group 1 according to the widely used classification of hydrogenases [28].

After in vivo H_2_ production in the wild-type and the mutant BL21(DE3) strains with *H. marinus* HoxGK, extracellular formate concentrations were measured and compared with those of negative controls (Table 1). All of the strains with the parent vector showed similar formate level regardless of the FHL mutations. This is not surprising because formate consuming pathways are already impaired in BL21(DE3) [11]. On the other hand, when *H. marinus* HoxGK was expressed, the formate concentration of wild-type BL21(DE3) was lower than those of the mutant strains, indicating that formate was consumed for H_2_ production. Notably, the overall formate level was lowered upon the expression of hydrogenase even in the mutants that cannot produce H_2_, which implies that formate metabolism (either production or consumption) other than FHL pathway was also affected by the expression of recombinant hydrogenase.

**Table 1:** Extracellular concentration of formate (mM) measured after in vivo H_2_ production in BL21(DE3) derivatives

Plasmid	Strain		
	Wild-type	*ΔfdhF*	*ΔhycE*
pTrcHis C	15.9 ± 0.3	16.5 ± 0.1	16.1 ± 0.4
pTrcHoxGK	10.6 ± 0.2	13.3 ± 1.1	13.1 ± 1.4

### Involvement of each subunit in FHL activation

**Fig. 3:**
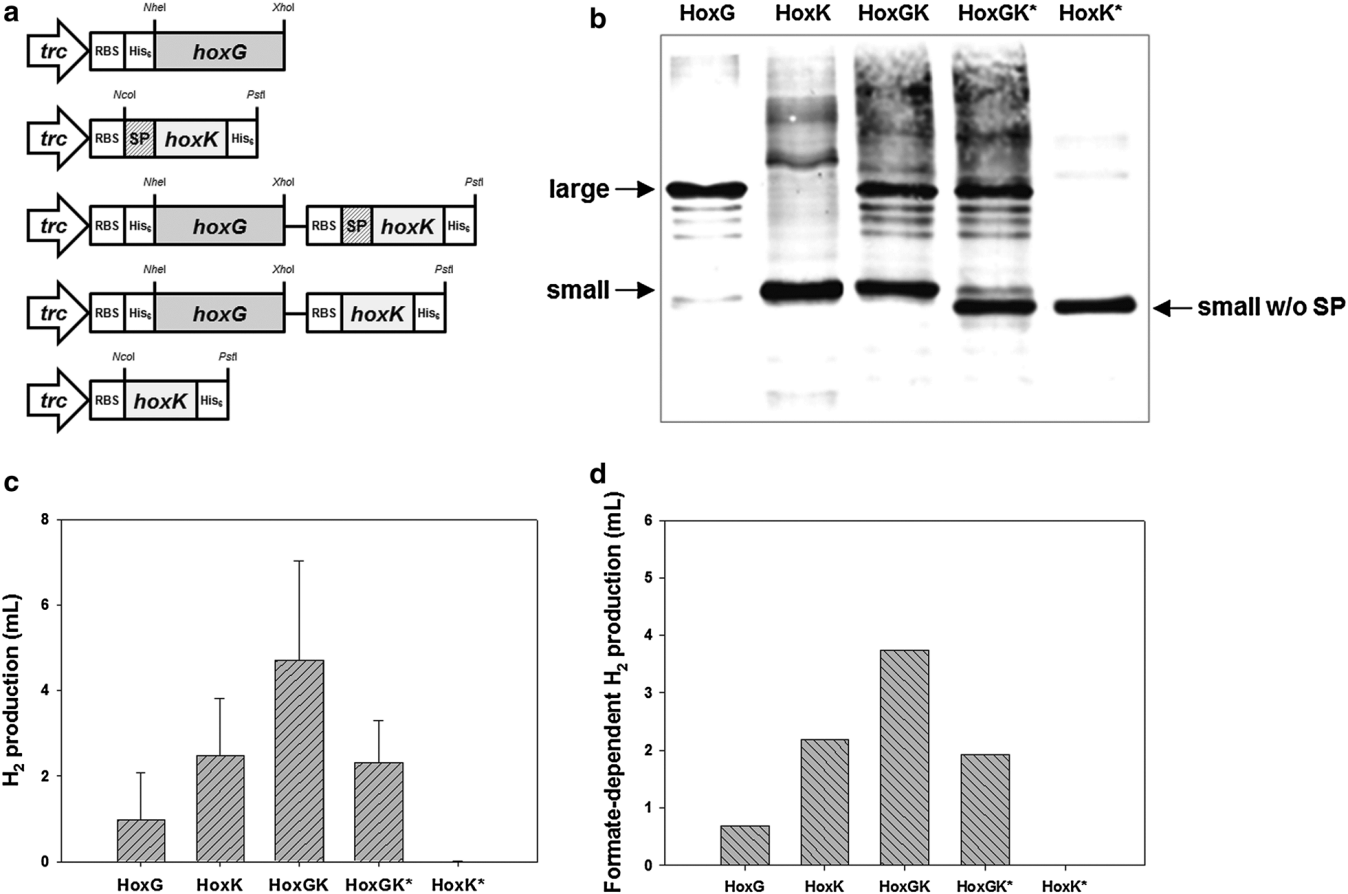
Combinatorial expressions of hydrogenase subunits of *H. marinus* in BL21(DE3). **a** Construction of expression vectors. The constructed vectors (from *top* to *bottom*) are pTrcHoxG, pTrcHoxK, pTrcHoxGK, pTrcHoxGK*, and pTrcHoxK*, respectively. **b** Western blot analysis. Anti-His_6_ antibody was used. **c** H_2_ production. **d** FHL activation. H_2_ production from formate was measured after 18-h incubation. *RBS* ribosome binding site, *His*_*6*_ hexahistidine tag sequence, *SP* sequence for signal peptide, HoxK* HoxK without signal peptide

In an effort to reveal the role of uptake [NiFe]-hydrogenase in FHL activation, we investigated the contribution of each subunit to H_2_ production using *H. marinus* hydrogenase as a model enzyme. Expression vectors were constructed for five different combinations of the large subunit (HoxG), small subunit (HoxK), and small subunit without signal peptide (HoxK*) (Fig. 3a), and all of the subunits with His_6_-tag were successfully expressed in *E. coli* BL21(DE3) (Fig. 3b). As shown in Fig. 3c, different amounts of H_2_ were produced by the different combinations. This pattern of H_2_ production was well correlated with FHL activity (*R*^2^ > 0.99) (Fig. 3d), implying that the different amounts of H_2_ production was due to different degrees of FHL activation. Intriguingly, H_2_ production was observed in the recombinant strains with HoxG or HoxK alone (Fig. 3c). Because the catalytic active site of [NiFe]-hydrogenase is located in large subunit [28], the result of H_2_ production with only the small subunit corroborates the previous conclusion that the recombinant hydrogenase was not the catalyst that produced H_2_ in BL21(DE3). Notably, the effects of the two subunits seemed to be additive (Fig. 3c), possibly representing the presence of more than two separate mechanisms for FHL activation. The fact that H_2_ was produced with HoxG alone also supports this possibility.

The deletion of signal sequence on HoxK resulted in no H_2_ production, indicating that the signal peptide was essential for FHL activation via the small subunit (Fig. 3c). This observation is consistent with the previous report, in which the importance of signal peptide on in vivo H_2_ production was shown [17]. Because the signal peptide is implicated in the interaction with membrane component(s) for protein translocation [29], it is likely that the mechanism by which the small subunit activates FHL involves a membrane component that directly or indirectly affects FHL, which is also a membrane protein complex [16].

A recent study on metabolic deficiencies of BL21(DE3) suggested that the lack of FHL activity in BL21(DE3) can be restored by complementation of a wild type copy of *fnr* gene and a high concentration of metal ions (500 μM nickel and 1 mM molybdenum) [11]. In our experiments, no additional ions were added except for 30 μM nickel and iron, and little possibility exists that the expressed subunits can function as FNR. Additionally, the effect of FHL restoration by FNR was only partial when compared with the FHL activity of *E. coli* K-12 strains [11]. Intriguingly, an *fnr* mutant of K-12 strain (PB1000) still possessed 20 % FHL activity of the parent strain [11]. Thus, although we do not offer any clear explanation of how the subunits activate FHL, we suggest the existence of an unknown pathway(s) for FHL activation and regulation of formate metabolism that is distinct from the *fnr*-mediated activation.

### Implications for future research

The main purpose of engineering hydrogenase or its relevant pathway is to enhance H_2_ yield and/or productivity. Because H_2_ production in the recombinant BL21(DE3) strains almost entirely depends on native FHL, in principle, the yield cannot exceed the theoretical maximal H_2_ yield from formate (2 mol-H_2_/mol-glucose) that has been almost realized with *E. coli* K-12 mutant (Table 2). In terms of productivity, the recombinant strains are also much less effective than previously constructed K-12 derivatives (Table 2). Therefore, in their present form, the reported BL21(DE3) strains with the recombinant uptake [NiFe]-hydrogenases appear to be poorly suited for practical in vivo H_2_ production unless non-native FHL-independent H_2_ pathways are constructed with the recombinant hydrogenases using synthetic biology and/or metabolic engineering approaches. Thus, we suggest that recombinant hydrogenase systems designed for in vivo H_2_ production should be carefully characterized, especially if *E. coli* BL21(DE3) is used as a host; mere observation of in vivo H_2_ production doesn’t imply successful construction of non-native H_2_ pathway.

**Table 2:** Comparison of H_2_ production by *E. coli* strains

Host	Genetic modification	H_2_ yield (mol-H_2_/mol-glucose)	H_2_ productivity (mL-H_2_/L-culture h)	References
*E. coli* BL21(DE3)	*H. marinus* *hoxGK*	0.65	25.1	[17]
*E. coli* BL21(DE3)	*R. sphaeroides* *hupSL*	0.28	19.7	[18]
*E. coli* BL21(DE3)	*E. coli hyaBA*	0.32	12.5	[19]
*E. coli* BL21(DE3)	*R. palustris* *hupSL*	0.32	39.9	[27]
*E. coli* BW25113	*ΔhycA ΔhyaAB ΔhybBC ΔldhA ΔfrdAB*	1.80	420.7	[30, 31]
*E. coli* BW25113	*ΔhyaB ΔhybC ΔhycA ΔfdoG ΔfrdC ΔldhA ΔaceE*	1.32	354.8	[32]

*E. coli* BL21(DE3) is an important strain as a general choice for overexpression of recombinant proteins [5] and holds promise for metabolic engineering and biofuel production. Complete elucidation of the mechanisms for FHL activation in BL21(DE3) is important because it could enable the efficient expansion of H_2_ yield with high productivity in *E. coli*; H_2_ might be produced using more than two substrates simultaneously in BL21(DE3) e.g., via the fully activated FHL pathway and the other FHL-independent H_2_ pathway that is robustly constituted by recombinant overexpression of H_2_ metabolizing enzymes [8].

## Conclusions

In this study, the H_2_ production pathway was investigated in recombinant *E. coli* BL21(DE3) strains that express the structural subunits of uptake [NiFe]-hydrogenase from *H. marinus* (HoxGK), *R. sphaeroides* (HupSL), or *E. coli* (HyaBA). The recombinant strains clearly showed FHL activity, whereas the wild-type strain did not. The H_2_ production was not observed in the recombinant strains lacking *fdhF* or *hycE*, thus demonstrating exclusive dependence of the H_2_ production on activated native FHL. Formate level was changed upon expression of hydrogenase even in the mutant strains lacking FHL, indicating that formate metabolism other than FHL was also affected. Through combinatorial expression of hydrogenase subunits, it was shown that each subunit could activate FHL independently. In addition, it was revealed that the signal peptide is required for FHL activation by the small subunit. The FHL dependence of the recombinant BL21(DE3) derivatives fundamentally limits the practical use of the strains in applications for biohydrogen production. A more effective system might be constructed by synergetic combination of an overproduced synthetic H_2_ pathway with the fully activated FHL pathway in *E. coli* BL21(DE3).

## Methods

### Strains and plasmid construction

The strains, plasmids, and primers used in this study are listed in Table 3. All of the DNA works were performed using *E. coli* TOP10 (Invitrogen, USA), and *E. coli* BL21(DE3) (Novagen, USA) was used for hydrogenase expression and H_2_ production. The plasmid for expression of *Rhodobacter sphaeroides* HupSL (pEMBTL-HJ2) [18] and the *E. coli* mutant strain MW1001 [33] were kindly provided by Dr. Jiho Min (Chonbuk National University, Jeonju, Korea) and Dr. T. K. Wood (Texas A & M University, Texas, USA), respectively. The vectors for expression of the hydrogenase subunits of *Hydrogenovibrio marinus* [34] were constructed by polymerase chain reaction (PCR)-based cloning procedures using genomic DNA of *H. marinus* (DSM 11271) and the listed primers with *Nhe*I, *Nco*I, *Xho*I, or *Pst*I restriction sites. The PCR products were inserted into the pGEM-T Easy vector (Promega, USA) prior to subcloning into pTrcHis C (Invitrogen). For polycistronic expression of both hydrogenase subunits, the primers hoxK_poly and hoxK*_poly were designed to contain an intergenic sequence with a ribosome binding site (RBS), a slightly modified portion of the intergenic sequence between *lacZ* and *lacY* found in the *E. coli* genome. The plasmid pTrcHoxGK was primarily used throughout the study for expression of *H. marinus* hydrogenase. *E. coli* cells were grown and maintained in Luria–Bertani (LB) medium (Usb Corp., USA) supplemented with the appropriate antibiotics (ampicillin, 50 μg/mL; streptomycin or kanamycin, 10 μg/mL) at 37 °C in a shaking incubator at 220 rpm (Jeiotech, Korea).

**Table 3:** *E. coli* strains, plasmids, and primers used in this study

Strains, plasmids, or primers	Genotypes, relevant characteristics, or sequences	Source or references
Strains		
TOP10	F^−^ *mcrA* *Δ*(*mrr*-*hsdRMS*-*mcrBC*) Ф80*lacZΔM15* *ΔlacX74* *recA1* *araD139* *Δ*(*ara*-*leu*) *7697* *galU* *galK* *rps*L (Str^R^) *endA1 nupG*, streptomycin-resistant	Invitrogen
BL21(DE3)	F- *ompT hsdS*_B_(r_B_^−^ m_B_^−^) *gal dcm* λ(DE3), carrying the T7 RNA polymerase gene	Novagen
JH0	BL21(DE3) *ΔfdhF::FRT*-*kan*-*FRT*	This study
JH1	BL21(DE3) *ΔhycE::FRT*-*kan*-*FRT*	This study
MW1001	*lacI* ^*q*^ *rrnB*_T14_ *ΔlacZ*_WJ16_ *hsdR514 ΔaraBAD*_AH33_ *ΔrhaBAD*_LD78_ *ΔhyaB ΔhybC ΔhycE*	[33]
Plasmids		
pGEM-T Easy	*bla lacZ*, TA cloning vector	Promega
pEMBTL-HJ2	Expression vector with T7 promoter carrying *R. sphaeroides* *hupS* and *hupL*	[18]
pTrc-EcH1ABHis	Expression vector with *trc* promoter carrying *E. coli hyaB* and *hyaA*	[19]
pTrcHis C	pBR322 ori *bla* *lacI* ^*q*^, a parental expression vector with *trc* promoter	Invitrogen
pTrcHoxGK	pTrcHis C carrying *H. marinus* *hoxG* and *hoxK*	This study
pTrcHoxG	pTrcHis C carrying *H. marinus* *hoxG*	This study
pTrcHoxK	pTrcHis C carrying *H. marinus* *hoxK*	This study
pTrcHoxGK*	pTrcHis C carrying *H. marinus* *hoxG* and *hoxK* without signal sequence	This study
pTrcHoxK*	pTrcHis C carrying *H. marinus* *hoxK* without signal sequence	This study
pKD46	*bla γ β exo araC*, Red recombinase vector containing temperature-sensitive replicon	CGSC
pKD13	*bla* *FRT*-*kan*-*FRT*, template plasmid used for Red recombination	CGSC
Primers^a^		
hoxG	Forward: GCTAGC **ATGAGCGTATTAAACACACC** (*Nhe*I) Reverse: CTCGAG **TTATCGAACCTTGACGGT** (*Xho*I)	This study
hoxK_poly	Forward: CTCGAGTCTGCCCGTATTGCGCGTAAGGAAATCCATTATG**TCAT CTCAAGTTGAAAC** (*Xho*I) Reverse: CTGCAGTCAATGGTGATGGTGATGATGACCGCC**TTTATCTCCTT TCTTTTGA**GCC (*Pst*I)	This study
hoxK*_poly	Forward: CTCGAGTCTGCCCGTATTGCGCGTAAGG**AAATCCATTATGGCG AACAAAATTGCTCATGCGAT** (*Xho*I) Reverse: *ditto*	This study
hoxK	Forward: CCATGGGC**TCATCTCAAGTTGAAACGTT** (*Nco*I) Reverse: *ditto*	This study
hoxK*	Forward: CCATGGGC**AACAAAATTGCTCATGCGAT** (*Nco*I) Reverse: *ditto*	This study
fdhF13	Forward: CAATCACGTACTGCTCGGCGGCGCGCTGATCGGCGATCGGCTCG ACGCGC**ATTCCGGGGATCCGTCGACC** Reverse: TCCTGACCCCGCGCCTGAAAACCCCCATGATCCGTCGCCAGCGT GGCGGC**TGTAGGCTGGAGCTGCTTCG**	This study
hycE13	Forward: TTTTTGATAAAGGTAAACATGGCGATTCCTTATTTCAGCGGCGA GTTTTT**ATTCCGGGGATCCGTCGACC** Reverse: TTAGCGTTCGTCTCCTTGCTGGCGGCGTGATTAAAGAGAGTTTG AGCATG**TGTAGGCTGGAGCTGCTTCG**	This study
fdhFchk	Forward: **GTAGGGAGTAACCAGTATAA** Reverse: **AATGACCCCACATAAAATGT**	This study
hycEchk	Forward: **CCAGCGGATAAGACGAGGT** Reverse: **CGTCTTGATATTACTCCGCG**	This study

^a^Regions that hybridize to the corresponding template sequences are bolded, and restriction sites are underlined

### Construction of mutant strains

The Red recombination system with pKD46 (Coli Genetic Stock Center (CGSC), USA) was adopted for inactivation of chromosomal *fdhF* or *hycE* gene in *E. coli* BL21(DE3). A gene construct composed of kanamycin resistance gene (*kan*) flanked by FLP recognition target (FRT) sites on pKD13 (CGSC) was amplified by PCR using *fdhF*- or *hycE*-specific primers with 50-nt homology extensions. Gene disruption was performed as described in [35] and confirmed by PCR using specific primers that were designed based on the sequences flanking the disrupted region of the genome. The *kan* gene was not cured to avoid contamination in cell culture.

### In vivo H_2_ production

The recombinant *E. coli* BL21(DE3) derivatives transformed with the expression vectors were cultured in 100 mL of M9 media (6 g/L Na_2_HPO_4_, 3 g/L KH_2_PO_4_, 1 g/L NH_4_Cl, 0.5 g/L NaCl, 2 mM MgSO_4_, and 100 μM CaCl_2_) supplemented with 5 g/L of casamino acids (BD Bioscience, USA), 5 g/L of glucose, and 50 μg/mL of ampicillin (and 10 μg/mL of kanamycin only for mutant strains) in 165-mL serum bottles (Wheaton, USA) at 37 °C and 220 rpm. When the cell density reached ~0.6 OD at 600 nm, the cultures were induced for hydrogenase expression and H_2_ production with the addition of 1 mM isopropyl-β-d-thiogalactopyranoside (IPTG; Carbosynth, UK), 30 μM NiSO_4_, and 30 μM FeSO_4_. The bottles were tightly sealed with rubber stoppers and aluminum caps and cultivated for a further 16 h until H_2_ production was measured using gas chromatography (GC; Younglin Instrument, Korea).

### FHL activity assay

After in vivo H_2_ production, cells were harvested by centrifugation at 4 °C and 4000×*g* for 10 min and washed with phosphate buffered saline (PBS; 8 g/L NaCl, 1.44 g/L Na_2_HPO_4_, 0.2 g/L KCl, and 0.24 g/L KH_2_PO_4_; pH 7.4). They were resuspended in 98 mL of PBS in the serum bottle with addition of 2 mL of 1 M sodium formate. Immediately after brief (~3 min) flushing with N_2_ gas, the bottle was sealed with a rubber stopper and an aluminum cap. After incubation at 37 °C and 220 rpm, the production of H_2_ from formate was analyzed from the gas phase of the bottle via GC.

### H_2_ production measurement

The H_2_ production was measured as previously described [36]. In brief, a specific volume (usually 100 μL) of gas was sampled from the headspace of culture bottle and analyzed by GC to determine the partial H_2_ pressure. The total amount of H_2_ was calculated by multiplying the H_2_ concentration by the headspace volume of the bottle (65 mL).

### Western blot analysis

Western blot analysis was performed for detection of hexahistidine (His_6_)-tagged proteins as previously described [36].

### Formate measurement

Formate was measured by enzymatic assay using formate dehydrogenase as previously described [37] with slight modifications. Samples were diluted 1/10 with deionized water. A reaction solution containing 610 μL of 80 mM sodium phosphate buffer (pH 7.0), 300 μL of 10 mM nicotinamide adenine dinucleotide (NAD^+^; Sigma-Aldrich, USA) and 100 μL of formate dehydrogenase (~1 mg/mL; Sigma-Aldrich) was mixed with 25 μL of the diluted sample solution. After 2.5 h reaction at 37 °C, the absorbance change by formate-dependent NAD^+^ reduction was measured at 340 nm. Formate concentration was calculated based on the absorbance change and a standard curve prepared using sodium formate solutions (Sigma-Aldrich) with various concentrations.
